# AttentivU: An EEG-Based Closed-Loop Biofeedback System for Real-Time Monitoring and Improvement of Engagement for Personalized Learning

**DOI:** 10.3390/s19235200

**Published:** 2019-11-27

**Authors:** Nataliya Kosmyna, Pattie Maes

**Affiliations:** MIT Media Lab, 75 Amherst St, E14-548, Cambridge, MA 02139, USA; pattie@media.mit.edu

**Keywords:** electroencephalography (EEG), feedback, closed loop, real-time, brain–computer interfaces

## Abstract

Information about a person’s engagement and attention might be a valuable asset in many settings including work situations, driving, and learning environments. To this end, we propose the first prototype of a device called AttentivU—a system that uses a wearable system which consists of two main components. Component 1 is represented by an EEG headband used to measure the engagement of a person in real-time. Component 2 is a scarf, which provides subtle, haptic feedback (vibrations) in real-time when the drop in engagement is detected. We tested AttentivU in two separate studies with 48 adults. The participants were engaged in a learning scenario of either watching three video lectures on different subjects or participating in a set of three face-to-face lectures with a professor. There were three conditions administrated during both studies: (1) biofeedback, meaning the scarf (component 2 of the system) was vibrating each time the EEG headband detected a drop in engagement; (2) random feedback, where the vibrations did not correlate or depend on the engagement level detected by the system, and (3) no feedback, when no vibrations were administered. The results show that the biofeedback condition redirected the engagement of the participants to the task at hand and improved their performance on comprehension tests.

## 1. Introduction

Everyday work and life are becoming increasingly complex and distractive, making it harder for people to show extended focus, attention or engagement on a specific task or lecture, thereby directly impacting their level of performance in everyday tasks.

**Figure 1:**
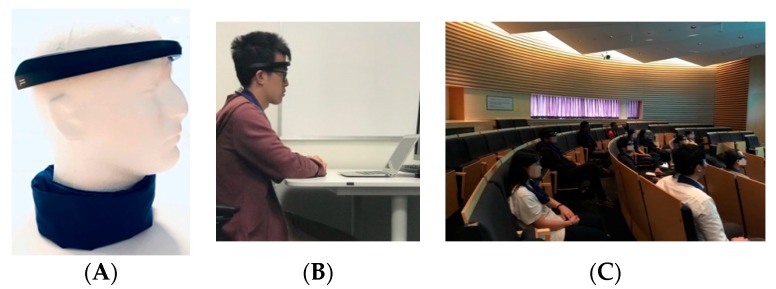
(**A**) Generic view of two main components used in AttentivU system: an electroencephalography (EEG) headband (Focus 1, BrainCo) and a blue cotton scarf, which contains two vibromotors used to provide a haptic feedback. (**B**) A person wearing the AttentivU system during Study 1. (**C**) Twelve participants wearing 12 units of AttentivU system during Study 2.

Several recent papers from human-computer interaction (HCI) conferences and journals, as well as signal processing and biomedical venues [1,2,3,4,5,6,7] explored measuring engagement using physiological sensors such as electroencephalography (EEG) in order to augment learning activities, for example, to provide information about one’s engagement to a teacher or to a presenter. The feedback loop in these works, however, is rarely closed, as the information about the attention or engagement level is not presented to the person themselves. In this work, we address this issue in the context of learning situations as we believe that real-time monitoring of engagement level and issuance of biofeedback might improve learning or work performance outcomes of the person. To this end, we propose the first prototype of a system called AttentivU. a wearable system which consists of two main components. Component 1 is represented by an EEG headband used to measure the engagement of a person in real time. Component 2 is a scarf, which provides subtle, haptic feedback (vibrations) in real time when the drop in engagement is detected (Figure 1).

Using 24 such devices, we tested our approach on 48 adults over several sessions in both lab and lecture settings. The results show that the biofeedback redirects their engagement to the task at hand and improves their performance on comprehension tests. We first discuss some related work to better position our contribution. We then describe the design and development of the AttentivU prototype followed by two studies we performed in order to evaluate its feasibility and effectiveness.

Regarding terminology, in the literature we find publications that use a variety of different terms, including vigilance, attention and engagement. Sometimes these are even used interchangeably. Engagement is a term with multiple definitions but the most common use for it is sustained attention or tonic alertness [8]. The term usually includes energetic arousal on the sleep–wake axis, intrinsic motivation, cognitive performance, and concentration according to Kamzanova et al. [9], Reinerman et al. [10], and Freeman et al. [11]. This paper defines engagement as the ability to focus on one specific task for a continuous amount of time without being distracted. Moreover, in our study, we follow a well-documented set of research works in order to increase the reproducibility of our work, as well as comparability to existing papers. For a more extensive discussion, we refer the reader to a review by Oken et al. [8] that includes a detailed discussion of different terms like “alertness”, “arousal”, “sustained attention”, and “vigilance”. We also recommend Freeman et al. [11] and Kamzanova et al. [9] for more in-depth discussions of engagement measures using EEG.

## 2. Related Work

### 2.1. Defining and Measuring Engagement in Learning Environments

Engagement in learning environments can be characterized as “a multidimensional construct with behavioral, emotional, and cognitive dimensions” [12]. Attendance and participation in school activities are examples of behavioral engagement. A sense of belonging or valuing of the school represents an emotional engagement. Willingness to engage in effortful tasks, purposiveness, and self-regulation are examples of cognitive engagement. Reeve and Tseng [13] proposed a fourth type of engagement, which they call agentic engagement, which occurs when a student contributes to the flow of instruction.

Shernoff et al. [14] proposed to identify student engagement based on concentration, interest and enjoyment.

Sinatra et al. [5] proposed to distinguish the “grain size” of engagement, the level at which engagement is conceptualized, observed, and measured, and they presented it as the framework “continuum of engagement measurement”. It could either be measured on individual “microlevel”, which represents engagement in the moment, task, or learning activity to a group “macrolevel”, which represents a group of learners in a class, course, or school. Microlevel engagement can be measured using brain imaging, skin conductivity or eye tracking. Macrolevel indicators of engagement could include observations, ratings, or other analyses of the sociocultural contexts of learning or schooling.

We further refer the reader to Sinatra et al. [5] and Azevedo [15] as well as their special issue on behavioral, group engagement in learning.

### 2.2. Measuring Individual’s Engagement Level Using EEG

We position our work with respect to the framework “continuum of engagement measurement” proposed by Sinatra et al. [5] discussed above. We measure cognitive engagement at the fine grain size, i.e., microlevel—an individual’s engagement in the moment, task, or learning activity.

Several technologies are currently available for monitoring user engagement with computer interfaces, including gaze- and eye-tracking systems and video recording devices, although these solutions are prone to errors and limitations. For example, the use of cameras in the classroom is against the policy of some schools and universities, thus rendering video tracking impossible.

Hutt et al. [4] used a set-up with a computer and $90 eye-tracker per individual student, but this solution is complex to set up as well as expensive (both a computer and a tracker are required for each user). In addition, they reported facing calibration failures and limited accuracy of the eye-tracker. Raca et al. [16,17] reported that head measurements alone were not enough to reliably estimate person’s attention level.

Other measures at this end of the continuum include physiological sensing such as heart-rate variability [18], galvanic skin response [19], and EEG [20]. Of those, we chose EEG as the basis for our engagement monitoring technique due to the numerous studies which showed that EEG signals can be used to identify subtle shifts in user alertness, attention, perception, and workload in laboratory, simulation, and real-world contexts [21,22]. EEG-based engagement measures were used to provide audience feedback [1] to a presenter and to log the engagement of workers in a simulated office condition [23]. Numerous studies further investigated the use of EEG-based engagement measures to augment learning activities. For example, the system “Pay Attention” [24] describes an embodied story-telling agent which uses modulated spoken volume and gestures to attract users’ attention when their engagement level drops. “Focus” [25] monitors a child’s engagement level in real time, and provides contextual brain–computer interface (BCI) training sessions to improve a child’s reading engagement. “Let’s Learn” by Andujar and Gilbert [7] is another EEG-augmented reading system, similar to “Focus”, which uses content-related videos from YouTube to improve users’ engagement, although no formal study was performed to validate the system. Finally, “Bravo” [26] estimates user’s attention and meditation levels and presents users with learning material that results in high engagement.

### 2.3. Delivering Feedback about Engagement Level

Very few studies deliver real-time feedback about the engagement level to the person, and existing ones are mainly proposed in the context of a user manipulating an interface, such as driving a car or playing a video game where an object would accelerate if a person is more focused [27]. However, providing immediate feedback to the user while they are on the task at hand might be engaging for students as suggested by Shernoff et al. [14] or Sclater et al. [28]. The closest system to the one proposed here is the reminder device Re:Vibe [29], which is a commercial wristband that uses vibrotactile stimuli delivered at random periods of time. However, it is not based on a scientifically validated approach on the effect of the vibrations on engagement. To the best of our knowledge, no research was done regarding delivering real-time biofeedback about engagement level based on biological data in a classroom setting. Two commercial EEG-based devices with no history of publications were launched recently. The first one, MindSet [30], is a device in the form of ear-covering headphones, thus making its use impossible in the classroom. Another one, by the recent start-up BrainCo [31], provides real-time feedback to the teacher about the attention of the students who wear EEG headbands; however, reports or studies are yet to be published.

## 3. Materials and Methods: Study 1

### 3.1. AttentivU: Design Choices and Implementation

#### 3.1.1. Form Factor for Engagement Measurement Component

We decided to use a lightweight, commercial EEG headband, similar to those used in other studies that measure engagement [1,7,23]. Using a band facilitates the reproducibility and generalizability of the study as well as its comparability to previous work. Because of the headband form factor and the sensitivity of EEG to noise, the system is not reliable when the wearer is walking [32]. However, as we target people who sit in a classroom, office, or a desk at home, we believe that this limitation is acceptable. We discuss the processing of EEG signals in the next section.

#### 3.1.2. Form Factor for the Feedback Component

For this project, we decided to use vibrotactile feedback. It is possible that visual and auditory feedback will prove to be overwhelming for a student who is surrounded by 15–20 peers who may be talking, as well as a teacher who the student has to pay attention to. However, visual and auditory modalities for feedback delivery might be considered as potential candidates for personalized learning experiences to be investigated in more depth in future studies.

We selected a scarf as a current form-factor for the feedback component based on the prior research which has identified the upper chest area below the collar bone and the wrist as the most optimal body locations for effective vibrotactile stimulation [33,34]. We also wanted to standardize haptic feedback delivery across users and avoid confusion with commercial wrist bands which use vibrations as a mean of notifications. However, while the scarf is used for the studies, it is not intended to be the final form factor.

**Figure 2:**
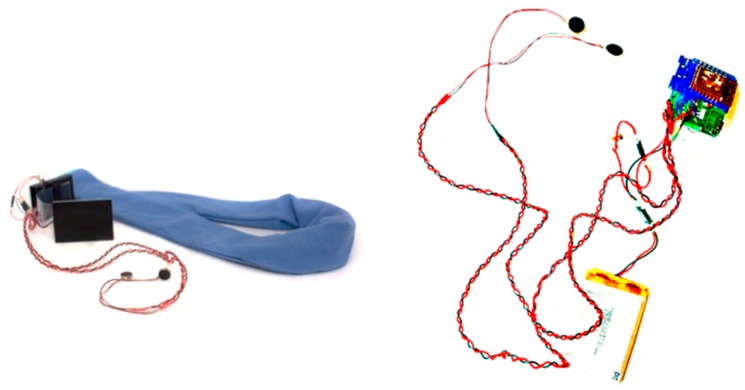
The scarf used in the study to provide the feedback to the users. (**Left**). The general view of the scarf. It is made of a soft blue fabric and contains a 3D case (in black) with all the electronics. Two motors are positioned outside the case. (**Right**). Electronic components of the scarf.

The scarf was made of a soft, cotton fabric (Figure 2, left). The full description of the scarf can be found in [35]. The scarf has a 3D case with an Adafruit Feather M2 Wi-Fi Arduino board and a 1000 mAh LiPo battery for a full day of use (Figure 2, right). The two vibration motors were located outside of the 3D case but inside of the scarf in order to avoid creating noise within the plastic case. The motors are positioned below the collar bones once the scarf is placed around the neck. The vibration lasted for one second. In order to design subtle haptic stimuli, the low intensity of 0.3 g (40 Hz; 1 g = earth’s gravitational field) was chosen and considered as appropriate and private. We did not experiment with different vibration patterns in this study.

#### 3.1.3. Engagement Index and Signal Processing

Pope et al. [36] used the “engagement index” to measure changes in engagement in participants while they completed cognitively demanding tasks. The engagement index E is calculated as

E = β/(α + θ),
(1)

where α (7–11 Hz), β (11–20 Hz), and θ (4–7 Hz) are neural oscillations that can be measured using EEG. This index was built assuming that an increase in beta power was related to an increase in brain activity during a mental task [19]: in fact, the beta frequency band could be associated with a number of cortical contributions like visual system activation, motion planning activity and other cortical functions mainly linked to an attentive state of the brain [36]. By contrast, increases in alpha and theta activity are related to lower mental vigilance and alertness. In particular, the presence of a synchronization in the alpha rhythm could be associated with periods of rest [37,38].

Our system measures engagement index ratio E, which represents the changes in the brain activity of the user related to reduction of cognitive activity in a task at hand (task could be external like an observation or internal—like imagination). The index E was tested in multiple set-ups and tasks, for example, a multi-attribute task battery (MATB) which includes tracking, monitoring, resource management, and communication tasks [36]; cognitive load [11], and visual processing and sustained attention [21]; and can identify changes in attention related to external stimuli [1,23].

The engagement index also measures vigilance and alertness, which are required in a learning process at the micro grain (individual) scale. A variety of experiments used this approach as a way to measure engagement and attention-related features [4,7,11,24,26,39].

**Figure 3:**
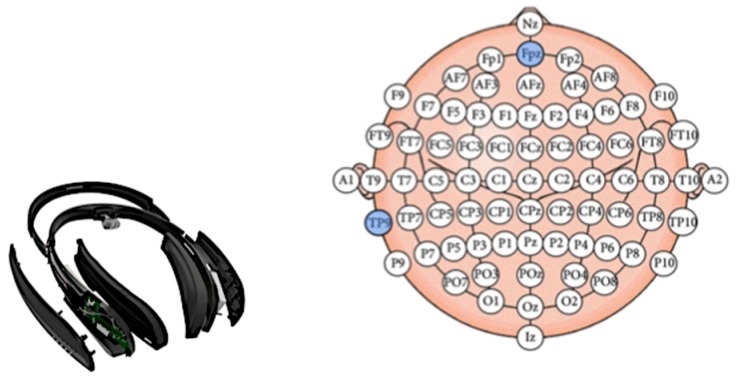
(**Left**). Focus 1 EEG headband, BrainCo used in this study. (**Right**). Electrode locations of Focus 1 band according to 10/20 System.

We based our system on prior research that reported using consumer EEG headbands with 1 to 6 channels. They are currently being widely used to detect cognitive engagement in the learning domain [1,4,7,24,26], as well as in other domains [6,21,40]. We use the BrainCo headband called Focus 1 (Figure 3, left), a lightweight EEG device with 3 hydrogel electrodes [35]. An electrode is located at the FPz position as well as reference and ground electrodes at TP9, according to the International 10–20 system of electrode placement (Figure 3, right). The headband collects the data at 160 Hz via Wi-Fi. An application for Mac that connects to the headband via Wi-Fi was used to collect and process EEG signals. We followed the signal processing pipeline proposed and presented in [1,24]. Raw EEG data was firstly notch-filtered with a central frequency of 60 Hz with a bandwidth of 10 Hz to remove power-line electrical disturbances and then with a band-pass filter of 4 to 20 Hz (because the starting frequency of θ band was 4 Hz and upper limit of β band was 20 Hz). We further down-sampled the filtered EEG followed by another pass through the same band-pass filter to avoid noise. The type of all filters used was IIR Butter-worth. Filtered EEG was then mean-shifted and divided by a constant scaling factor of 8. We then clipped the normalized, filtered EEG spikes that exceeded the amplitude range [−4,4] in order to avoid K-complex like spikes caused by minor external motion artefacts.

Our engagement index E is modeled from Pope et al. [36], in which we input the averaged power of alpha, beta, and theta frequency components obtained from the Power Spectral Density (PSD) over 5-second sliding windows. Next, we smooth E using an Exponentially Weighted Moving Average. This picked up general engagement trends and further removed movement artefacts. This outputs a smoothed engagement index per 15 seconds E*smooth* sent to the application.

A frame of 15 s was chosen after pretesting to identify minimum timeframe that grants sufficient data to accurately make predictions and also goes in line with the previous literature, like in [24].

The smoothing duration was chosen empirically after the pilot study to identify a minimum timeframe that provides sufficient data to accurately make predictions, as was done in [24].

Participants first went through a calibration phase, then a recording phase. The calibration session determined the distribution of E from E*min* to E*max*, where E*min* is low engagement (e.g., relaxation) and E*max* is high engagement (e.g., solving arithmetic problems) [1]. Please see User Studies section for further details. Based on the minimum E*min* and maximum E*max* engagement scores collected from the calibration for each participant, we calculated a normalized engagement score for each participant between 0 and 100 as:

E*norm* = (E*smooth* − E*min*)/(E*max* − E*min*) × 100 (2)

in a similar fashion to [1,35,39,40].

We divided the engagement index into low, medium, and high levels of engagement in a similar fashion to [1,35,39,40]. An engagement score of 0–30 is considered as low, 31–70 as medium, and 71–100 as high. The haptic feedback in the scarf is activated (e.g., sends a vibration) only when engagement is considered to be low for at least 15 s (empirically determined duration accounting for the possible false positives in the classification output).

### 3.2. Pilot Studies

#### 3.2.1. Pilot Study 1

Before the actual studies took place, we conducted a small pilot study where we asked three participants (two males, one female ages 22 to 27 years old) to watch a 25 min video lecture on fast Fourier transforms (FFT). The pilot test was performed to evaluate whether the engagement index E was indeed capable of identifying moments of focused engagement versus mind wandering.

All of the participants were aware of FFT but none of them knew the mathematical foundation of FFT. The lecture contained mathematical formulas and definitions. Participants did not wear the feedback component of AttentivU but only the EEG headband. Prior to the start of the video, we calibrated the system by asking participants to perform six sets of arithmetic tasks. Each participant was presented with 10 arithmetic expressions, adding two two-digit integers to one another, and entering their response in a text-box. Participants were also given Logitech clickers which they were supposed to use each time they feel that they start mind-wandering. We used this task set as a calibration task, as the lecture was also heavily computational and mathematical; thus, we hoped to induce similar states to those the user might experience during the main task of watching the FFT video. This type of task is used in several research setups, for example, by Hinks et al. [41]. Once a session was completed by a participant, a new one started. All the sessions contained the exact same arithmetic expressions (e.g., 32 + 19 would appear in each of the six sets); their order was, however, randomized. We expected to induce state from high to low engagement with such a set-up. We asked the participants to work as fast as possible, though we did not impose any time constraints. Once all 6 sets of tasks were completed, the video lecture on FFT started. We instructed the participants to use the Logitech clicker in the same manner as during the baseline task. We also told them that four different audio instructions would be played during the lecture and we asked them to perform the instruction they would hear but to try and keep their focus on the video lecture at the same time. By doing so, we were interested in testing whether the system would be able to detect the shift in their engagement. The instructions were provided as follows: “*think about your last conversation with your family”; “think about a current work challenge you are facing”; “think about a bird you saw recently”;* and “*think about anything that crosses your mind*”.

**Figure 4:**
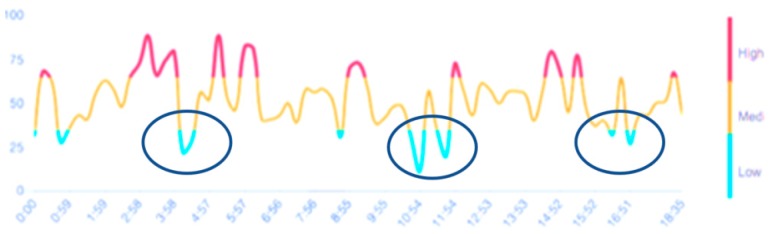
Engagement scores of participant 3 in Pilot Study 1. Low engagement scores were detected at the 4th, 9th, as well as 11th and 12th minutes (encircled).

The engagement score distribution for participant 3 is provided as an example on Figure 4. The results were similar for the other two participants. Low engagement scores in the 4th, 9th, as well as 11th and 12th minutes indicate a 15 s onset after the audio instruction was played to the participant.

#### 3.2.2. Pilot Study 2

We next performed a second pilot study to check whether we were able to successfully record the brain activity of several participants at the same time in a classroom setting, while a lecturer was presenting a live 25 min lecture, in this case about deep learning. Nine participants (five males, four females, ages from 22 to 29, different from the participants in Pilot Study 1) participated in this second pilot study and were not given any vibration feedback when their engagement dropped. This was a simultaneous multiuser study in a classroom set-up where all the participants were sitting in front of a big white board where the slides were projected. This study took place in the same room as our main Study 2. We informally collected observations and opinions of the participants on how they felt and how they perceived themselves as well as their peers wearing the headset during the lecture. All the participants reported that the experience was normal and that the they did not feel particular discomfort while wearing the headset. We also checked if the recorded data were received reliably via Wi-Fi in real time and saved correctly.

#### 3.2.3. Pilot Study 3

Finally, 9 participants (five females, four males, ages ranging from 25 to 30 years old, different from the participants in pilot studies 1 and 2) were invited to participate in our pilot Study 3. We were interested in validating our choices for intensity and duration of the vibration feedback during this final pilot study. During a 35 min period, each of the participants was invited into an office-like room to watch a YouTube video on the future of Internet of Things (IoT) on a Macbook Air 13” placed on the desk. Participants sat comfortably in the chair approximately 50 cm from the desk. Five types of vibrations of varying intensity (starting with 0.3 g; everything below 0.3 g was not perceivable during initial set-up) were administered to the participant every 7 min of the video duration. This study took place in the same room as our main Study 1. The participants reported which vibrations they felt as noticeable but not bothersome. All participants evaluated a low intensity vibration of 0.3 g as noticeable and least bothersome on a scale from 1 to 5. We thus used this intensity in our main studies.

### 3.3. User Study 1: Methods and Measures

Though we did not identify the same approaches as the ones used in our work, based on the works of Shernoff et al. [14] and Szafir and Mutlu [24] on immediacy cues in learning, where the system provided immediate feedback when the drop in engagement was detected, we propose the following three hypotheses in order to investigate the use of AttentivU in learning environments:

**Hypothesis** **1.** *Haptic feedback stimulation triggered by drops in EEG-monitored engagement levels will increase user’s engagement*.

**Hypothesis** **2.** *Haptic feedback triggered by drops in EEG-monitored engagement will positively affect participants’ evaluations of the system during subjective evaluation and interviews.*

**Hypothesis** **3.** *Haptic feedback will improve learning performance of users (retention and comprehension of presented information).*

As an important disclaimer regarding the learning performance in our studies: in this paper we mean “momentary learning performance”, meaning immediate results after the information was presented to a person, in contrast to a definition of the learning performance as measuring “learning gains over time” [42]. In our studies we did not perform tests again on the same content over a longer period of time; thus, all cases of learning performance we mention here are referred to as “momentary” ones.

To investigate the effect of haptic biofeedback in a learning setting, we designed and conducted two studies. The first study was laboratory-based, while the second one was a series of three lectures in a real lecture room with an actual lecturer. Both studies were approved by the ethical committee of Massachusetts Institute of Technology (protocol 1803282107A001). Participants were compensated with a $15 Amazon gift card per session.

#### 3.3.1. Participants

In total, 39 (21 males, 18 females) participants were recruited for Study 1, but three male participants were later excluded from the final analysis due to their excessive movements during the experiment (they did not commit to the requirement of the study to try not to perform unnecessary movements and were talking or standing during some moments of the study), thus leaving 36 (18 males, 18 females) participants with a mean age of 28. The participants were recruited through mailing lists distributed to the local research institution community as well as via a pool of prospective participants from the local cognitive lab, where 12 participants recruited from that pool were over 45 years old, and six had limited computer literacy.

#### 3.3.2. Experimental Task

The primary intended use case for AttentivU is the learning setting, more specifically, users absorbing new information presented either in the form of a video or through a live lecture. For this first study, we focused on videos and chose three different video lectures to be viewed by participants in the study. The lectures were on the following three subjects: neural networks (NN), Bitcoin and DNA cloning. We chose these three topics as they are often mentioned in the media these days and might be of some interest to participants, but they are also complex topics. All three lectures were inundated with formulas and technical terms, thus making parts of the lectures more challenging to follow. The NN lecture lasted 19 min, the Bitcoin lecture 26 min, and the DNA lecture 11 min. Lectures were narrative-style and did not include any quizzes in the middle of the videos. Each lecture was stand-alone and did not require any prior knowledge from the participants in order to be understood. The main task of the participants was to watch the lecture and pay attention as there would be questions regarding the content at the end of the lecture (Figure 1B). There were ten questions in increasing order of difficulty. The first three were multiple choice questions, while the remaining seven were open-ended. The questions were based exclusively on the content of each lecture, asking for lecture-specific examples and analogies. In total, 34/36 participants were novices in DNA cloning, 30/36 reported elementary knowledge of NN, and 24/36 participants reported having little knowledge about Blockchain.

#### 3.3.3. Experimental Procedure

Each participant arrived at a quiet study room with no windows and was briefed about the study before signing the consent form. The goals and the set-up of the study were explicitly mentioned in the consent form as well as repeated orally by the experimenter; each participant was expected to watch all three lectures and answer questions about the content of each lecture once each video lecture ended. Participants were instructed to stay as attentive as possible. Participants were told that the scarf may vibrate if a drop in engagement was detected. Participants were also briefed in very general terms about BCIs and about the headband they were about to wear. They were instructed about motion artifacts and were asked explicitly not to perform any unnecessary movements. Before starting the lectures, participants answered a background questionnaire, which included questions about their education, age, and their level of knowledge of NN, Bitcoins and DNA cloning. Once the background questionnaire was completed, participants put on the EEG headband as well as the scarf. The experimenter ensured that the vibrations were felt by the participants and that they did not bother them. The experiment started with a calibration phase. It consisted of three successive tasks similar to the ones used in [35]. Participants were asked to perform the following:

Task 1. Sit still with their eyes closed for 90 s.

Task 2. Sit still with their eyes open for 90 s.

Task 3: Alternate between 10 arithmetic and image-matching tasks (each lasting 30 s), with 10 s of controlled rest between each task. The image-matching task asked participants to indicate whether sequences of images match each other. This setup is similar to previous BCI work using n-back tasks [41], only here n equals 1 throughout the entire task, similar to the low cognitive workload condition used in previous BCI work [6]. We established high and low engagement for these tasks based on our engagement measure E. During the calibration stage users were explicitly instructed not to attend to any particular imagination or other internal process. This calibration phase lasted 5 min. EEG signals differ in each of these sessions (engagement was very low in Sessions 1 and 2, and slightly higher during Session 3 in the 1-back task, however the exact values were person-dependent).

For each of the calibrations tasks, the value of E over time in order to sample a representative distribution of engagement values for each participant was recorded. We used the characteristics of the distribution to determine a low engagement threshold for each user. Post-calibration, the experimenter moved out of the participant’s line-of-sight, and the participants started watching the lectures. The experimenter could see the participants and took notes if they were moved excessively. The order of the lectures was randomized for each participant. After the first video lecture was over, questions about that lecture appeared. Once the participant answered the questions about the lecture content, he/she filled out one more questionnaire–about the behavior of the scarf, which included questions like: “*How accurate was the feedback from the scarf?”, “How much were you able to retain the concepts from the lecture?”, “How engaged did you feel during the lecture?”, or “Do you think that the haptic feedback (vibrations from the scarf) helped you to focus (stay engaged)?*”.

After the participants finished both questionnaires, they had a two-minute break and a second video lecture started. After the second and third video lectures, there was once again a quiz unique to the content of the lecture and a general questionnaire about the behavior of the system (the questions about the system were always identical). Once participants watched all three lectures, a final questionnaire appeared on the screen; it contained several questions that were not related to the accuracy of the haptic feedback, such as “*If you were to wear this scarf in the social environment would you wear it again from time to time, when you felt like you needed it?*”.

After participants finished answering the final questionnaire, the experimenter helped them take off both devices. Also, the experimenter asked the participants for informal feedback about their experience. The study lasted around 1 h 45 min per person.

To test our hypotheses, we manipulated whether or not participants were receiving biofeedback from the scarf. There were three conditions:

(1) Biofeedback. Each time a drop in engagement from medium to low was detected, the scarf was set to vibrate. The vibration was administered within 60 s if the engagement level remained low after the first vibration was already administrated.
(2) Random feedback. The scarf would vibrate, but the vibrations did not correlate or depend on the engagement level of the user. The vibrations were set up in the following manner: No vibration was allowed within 2 min following random vibration; a vibration could occur in a time period of between 4 to 6 min (4 for shorter, 6 for longer lectures) to ensure that the vibrations were not too frequent/annoying.
(3) No feedback (NF). The scarf did not vibrate.

We recorded the brain activity of all the participants including the ones in the NF condition. To counterbalance our study, as there were three different lectures shown to each participant, we associated each lecture with a particular feedback type for each participant, which means that each participant experienced all three types of feedback during the experiment. In total, 12 participants received biofeedback on the NN lecture, 12 other participants were administered a random feedback on the NN lecture, and 12 different participants did not receive any feedback on the NN lecture, and so on. The experiment was double-blind. The dependent variables included engagement level of the participants, participants’ responses to the questions about the content of the lectures, as well as their perception of the system in subjective questionnaires.

## 4. Results of Study 1

We used a linear mixed model (REML fit at 742.4) on a within-participant basis (engagement ~ feedback + (1|participant) + (1|feedback:participant)) and then a repeated measures ANOVA on said model. The unit of measure was at the group level (feedback type). We had 108 observations with 36 participants. For the repeated-measures ANOVA, we had F(2) = 161.14, *p* = 2.2 × 10^−16^. Fixed effect (biofeedback) = 16.04; Fixed effect (no feedback) = −7.68.

We also tested for the influence of feedback type, lecture types, and potential order effects of lecture types. We received nonstatistically significant results from a uniformly randomized repeated measures ANOVA (F(2) = 5.33, *p* > 0.05).

**Figure 5:**
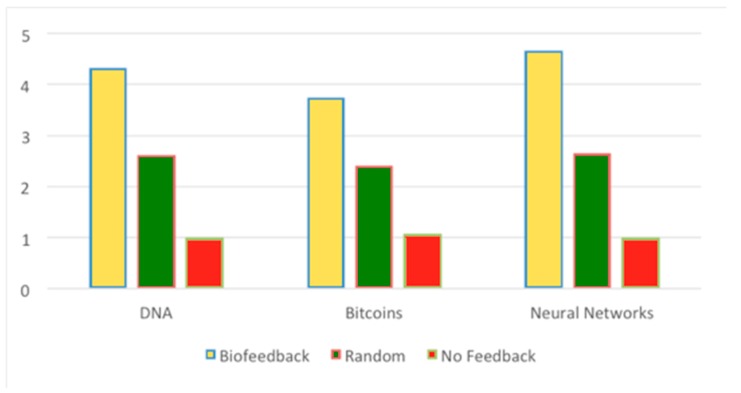
Average users’ response on their perceived engagement across different types of lectures (Likert scale 1–5 on *Y*-axis). Users reported being more engaged when biofeedback was provided to them compared to random or no feedback.

Figure 5 shows the average users’ response on their perceived engagement across different types of lectures (Likert scale 1–5). When we provided the biofeedback to the participants, they were more engaged as compared to random or no feedback. Participants who were provided biofeedback reported higher engagement (mean(M) = 4.52, SD = 0.23, standard error (SE). = 0.079) than participants who were provided random feedback (M = 2.38, SD. = 0.321, SE. = 0.107) or no feedback (M = 1.02, St.d. = 0.046, St.Err. = 0.015). This finding does not reject Hypothesis 2, whereby haptic feedback triggered by drops in EEG-monitored engagement positively affected participants’ evaluations of the system during subjective evaluation.

**Figure 6:**
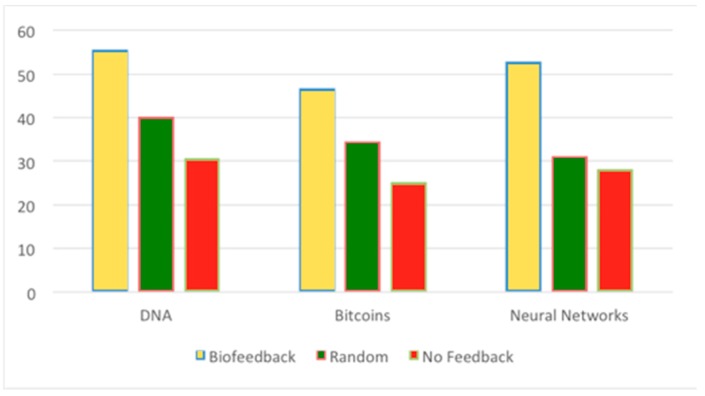
Average engagement scores (on *Y*-axis) computed from the EEG data across different lectures. Participants who received biofeedback were significantly more engaged compared to the random and no feedback conditions.

Participants who received biofeedback were significantly more engaged (M = 51.72, SD.= 4.51, SE. = 1.506) than participants who received random feedback (M = 35.32, SD. = 4.65, SE. = 1.55) or no feedback (M = 28.07, SD. = 2.69, SE. = 0.898). Figure 6 shows the average engagement scores computed from EEG data across three lectures combined for different feedback conditions. This finding does not reject Hypothesis 1, whereby haptic feedback stimulation triggered by drops in EEG-monitored engagement levels increased users’ engagement.

### 4.1. Performance on Content Tests

The contents tests contained questions which were designed and scored by the experimenters and researchers involved in the study, with the first four questions being easy, 0.25 point/question, and the last three questions being difficult, counting for two points/question, plus three questions of medium difficulty that counted for one point each. Participants who received biofeedback scored higher (M = 7.346, SD. = 3.16, SE. = 1.08) than participants who received random feedback (M = 5.27, SD. = 1.72, SD. = 0.601) or no feedback (NF) (M = 4.38, SD. = 0.91, SE. = 0.49). This finding does not reject Hypothesis 3, whereby haptic feedback improved momentary learning performance of users (retention and comprehension of presented concepts).

### 4.2. Design Insights from Questionnaires

We additionally administrated questionnaires about the form factor of the EEG band and the scarf. The scarf was appreciated by almost everyone (9/12). One user suggested developing a pendant instead of a scarf, another a brooch. We were also interested in the social acceptability of the system and the feelings about a possibility of using both devices in real life. The scarf was perceived as socially acceptable (4 points on the scale of 5 for 30 users). One user reported that it is “*ok for home use, personal use and if other people around me would wear the same thing—school or corporate style in a way.*”.

Regarding the EEG head band form factor, unsurprisingly, it got mixed reviews, but 6/36 users mentioned explicitly that they were aware of these bands from news outlets, and home use or group usage did not bother them provided the system helps. We discuss further developments on the form factor in the Future Work section.

### 4.3. Informal Interviews with Users

#### 4.3.1. Remarks on Feedback Effectiveness

Of the 36 users who participated in the studies, only two were concerned with the vibrotactile feedback and reported that it bothered them. The remaining participants appreciated the feedback they received from the scarf. For example, Participant 34 commented: “I liked it, this was actually pretty good. The buzzing was not loud, and it definitely helped me with staying more focused. It was scary how accurate it was. I was dozing off, and then this thing reminded me, and I was like, ** it, I am dozing off.”.

Participant 21, who received the order of conditions as biofeedback–random–no feedback, reported, “*I really liked it. In the first lecture, I decided not to pay attention on purpose, and it got me, it vibrated! But then in the last one, I tried to do the same thing and it really did not do anything at all. So I am guessing that all the lectures were having different set-ups for the buzzing feature.*”.

Participant 16, who also received the biofeedback–random–no feedback order of conditions, said, “*During the first lecture it was fine. As it was not my field at all and not of any particular interest, I was really losing attention, and it caught me. I think it did help me. But during the second and the third lecture it was behaving much worse. I am not sure if this is the part of the test, but on the third one it was not vibrating at all (“no feedback” condition), I did not feel anything.*”.

Participant 7, who received no feedback–random–biofeedback order of conditions, mentioned, “*the second and the third videos were really ok, especially the third one, it really felt very precise when I was getting the vibrations. The second video was kind of ok, though definitely not as precise as the third one.*”.

#### 4.3.2. Measuring Engagement

Five users were aware of the device and that it might have influenced their engagement level.

For example, Participant 29 reported that she was “*very conscious about the necklace even when it did not vibrate, I knew that it is there and that I am supposed to pay attention, so I told myself, ‘I should try to really pay attention,’ it was for me a presence thing, despite the vibrations being accurate, especially when I realized that I started being sleepy.*”.

Finally, several users like Participant 19, suggested use cases where they would use the system, “*I think it was more useful in some lectures than in other ones. I felt that during one of the lectures, it was really random, but then, during another one, I was starting to drift and...it worked, it buzzed! And I instantly thought that it would be awesome for classes, yes, please!*”.

#### 4.3.3. Conclusions for Study 1

Our first and third hypotheses, whereby haptic feedback will increase engagement and improve learning performance, were not rejected in the experiment. As seen from the results, the participants who received biofeedback demonstrated significantly higher EEG-based engagement scores than those who received either random or no feedback. Our second hypothesis, whereby participants would positively perceive the haptic feedback when evaluating the system, was also not rejected, based on the data collected from the questionnaires, informal interviews, and comments from the participants. It would be interesting to encounter the prior interest for the videos as well as the familiarity with the topics of the videos to gain even more insight for the designs of future studies. We further discuss the implications of this study in the Future Work section.

## 5. User Study 2: Methods and Measures

We next decided to perform another experiment, Study 2, in a set-up as close as possible to that of an actual classroom. The experiment involved multiple simultaneous participants during an actual, live lecture in a real lecture hall. This study was conducted to test the feasibility of deploying the system in conditions closer to a real-life learning scenario in a classroom, with some exceptions which are highlighted in experimental procedure.

### 5.1. Participants

For the second study, 12 participants (six males, six females, mean age 21 years old) were recruited from the students of a large university campus. All the participants were different from Study 1 participants. They were not familiar with the research or the AttentivU system.

### 5.2. Experimental Task

The set-up for this experiment was very similar to that of Study 1, with a few exceptions. It was a simultaneous, multiple participant study with all 12 participants present at the same time for three live lectures (Figure 1C). The lectures took place in a real university lecture hall over a period of three weeks (one per week). The three lectures were about different aspects of the same subject of virtual reality (VR), and they were presented in person by a lecturer who is an expert in VR. The lectures were related to each other and they were of increasing difficulty. Each lecture lasted between 40 and 50 min. Each lecture was presented by the lecturer with the help of a deck of slides. The slides included one short YouTube video per lecture, one live question from the lecturer to the students, one “live” demo from the lecturer illustrating one of the concepts of the lecture and blocks of slides loaded with mathematical formulas. The main task of the students was to attend the lecture and listen to the speaker.

As in the previous study, at the end of each lecture, participants had to answer 10 questions about the contents of the lecture. The questions were based exclusively on the contents of each lecture and they were lecture-specific. Each slide deck was designed by a researcher and the lecturer prior to the lecture taking place. Lecture 1 consisted of 56 slides, Lecture 2 had 50 slides, and Lecture 3 had 58 slides. The lecturer tagged each slide according to one of the following categories: slides were either labeled as “ok”, “boring”, “interesting”, or “exciting”. There was at least one interesting and exciting block (video or animations) of slides in each lecture. The slides were designed with the following strategy in mind: as engagement is a fluctuating state and it might not be possible to see some trends and changes right after a single slide, each slide category (“ok”, “boring”, and so on) was composed of at least 5–7 slides to establish that we could pick up the states of boredom and excitement. These annotations were based on the experience of the lecturer, a specialist in the domain. He knew about the purpose of the study and our interest in inducing varying levels of engagement throughout the lectures. The lectures were video-recorded.

### 5.3. Experimental Procedure

The experimental procedure for this study was similar to the previous one with a few exceptions. All participants were students at the university where the study took place. There was a spare seat in between each of the participants. Once all the participants arrived in the room, the researcher briefed them about the study before asking them to sign the consent form. Participants were asked to attend and listen to the lectures, which this time took place over the course of three weeks. Participants were not explicitly instructed to stay as attentive as possible, nor were they told that there would be questions to be answered after a lecture took place. They were asked to behave as naturally as they might in a regular class lecture. Regarding the scarf, the participants were told that it might vibrate, but no explanation was given to the participants about the reason why it might do so. Participants underwent the same procedure as in the previous study: They were given explanations about BCIs and motion artefacts, and they were asked not to perform unnecessary movements or restrain from taking notes. They filled out the background questionnaire (including questions on their education and level of expertise in VR), the devices were put on them by the experimenter, ensuring good connection, and finally they underwent a calibration phase (which lasted 5 min as in the previous study). Once the calibration session was over, the experimenter went to the other side of the room, where no participant was sitting, and the students started listening to the lecture. At the end of the lecture, participants were asked to fill out three questionnaires: a quiz on the contents of the lecture they just attended, their perception of the behavior of the scarf, where almost all the questions were similar to the ones in Study 1 (though some new ones were added, like, “*When did the scarf vibrate in your opinion?*”).

Finally, a third questionnaire was an actual deck of the slides of the lecture the students just attended, and the participants were asked to indicate for each slide whether they think they were engaged or not when that slide was used during the lecture. The slides were printed in a small preview format (16 slides per page) so participants would not really have the chance to review the content of the slides. Instead they just had a glimpse of what the slides were about. This was done so as not to bias their perception. Though we consider this method to not be perfect (as participants need to rely on their memory), we thought that it would be a better way to assess their subjective engagement score rather than using a clicker. Clickers tend to possibly help participants get back on track, but we did not want to bias the study by adding another device, and we wanted our lectures to be as similar to an actual lecture as possible. The second and third lectures were held in the same manner, with the exception that the background questionnaire was not administered. Calibration was performed before each lecture took place. After the third lecture, the participants got an additional questionnaire, called final, which was similar to the final questionnaire used in Study 1. Additionally, the participants were asked to share their feedback with the experimenter in a one-on-one conversation if they felt like it. Each lecture lasted approximately 1 h 45 min.

To test our hypotheses, we varied the type of feedback the participants obtained from the scarf. As in Study 1, there were three conditions. As there were 12 participants, 4 participants were assigned to get biofeedback, 4 other participants were assigned to receive random feedback (administered in the same way as in Study 1) and the last 4 participants did not get any feedback. Participants were assigned the same feedback type for all three lectures with no changes. Participants did not know which feedback was assigned to them, nor did they know that these three options existed. The study was double-blind. The dependent variables included participants’ engagement level, participants’ accuracy regarding their answers to the questions about the contents of the lectures, their self-reported engagement level, as well as their perception of the system in the subjective questionnaires.

## 6. Results of Study 2

As we only had 12 users in this study, we consider it to be more of a proof of concept of multi-user deployment of the system. The results of this study should be taken with precaution and they are only reported as preliminary as there are insufficient data to reliably estimate variances.

**Figure 7:**
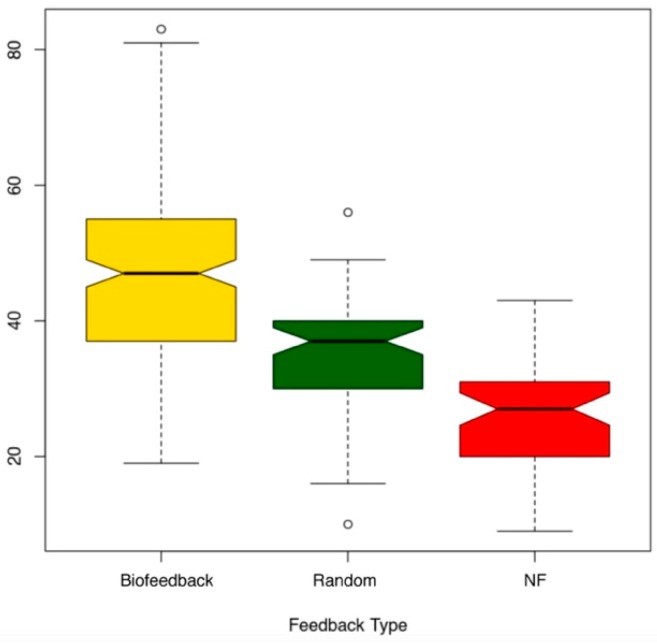
Engagement scores (on *Y*-axis) obtained for biofeedback, random and no feedback (NF) conditions for all three lectures, Study 2.

A linear mixed model (lmer4 package in R) analysis of variance (ANOVA) to compare engagement scores across feedback types was applied. We checked for normality with Shapiro-Wilk test (med chi-squared = 16.433, df = 2, *p* < 0.001), and for homogeneity of variances with Fligner–Killeen (W = 0.98834, *p* < 0.01), the fixed effect was the feedback type; random effects and parameters of the ANOVA are summarized in Table 1. We performed a pairwise post-hoc test on the mixed model with adjusted holm. We found significant differences for all pairs (*p* < 0.001 for all, see Table 1). The highest engagement scores were obtained from the biofeedback condition, followed by random feedback condition, followed by the no feedback condition (Figure 7). This finding goes in line with Hypothesis 1, whereby haptic feedback stimulation triggered by changes in EEG signals increased users’ engagement.

**Figure 8:**
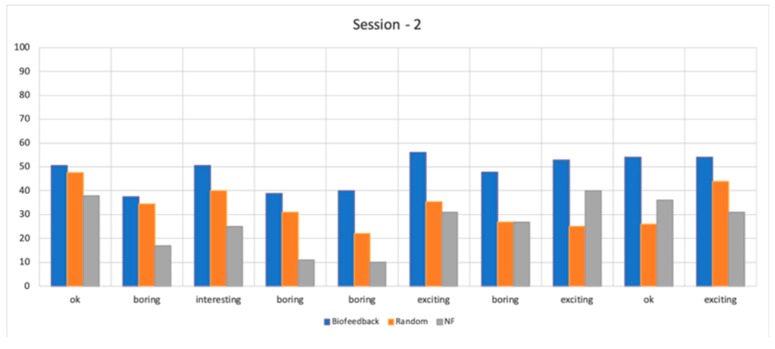
Average objective engagement scores (engagement index E on *Y*-axis) of participants during Lecture 2, Study 2. The *X*-axis represents slide type in the order in which they appeared (clusters of slides labeled as “ok”, “boring”, “interesting” or “exciting”).

Figure 8 presents average objective engagement scores of participants during Lecture 2. The *X*-axis of the figure represents slide type (clusters of slides labeled as “ok”, “boring”, “interesting”, or “exciting”).

**Figure 9:**
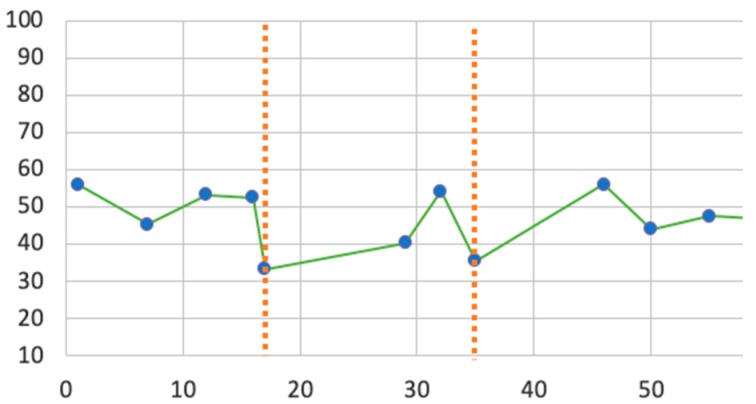
Engagement index E on *Y*-axis of Participant 2 during Lecture 1, Study 2, provided for illustrative purposes, as an example. The *X*-axis represents the slide number. The orange vertical line illustrates when the scarf of the participant vibrated (participant was a part of the biofeedback condition).

Finally, Figure 9 shows the average engagement index E of Participant 2 during Lecture 1. The *X*-axis represents slide type (clusters of slides). Orange vertical lines illustrate when the scarf of the participant vibrated (participant was a part of the biofeedback condition group).

### 6.1. Performance on Content Tests

**Figure 10:**
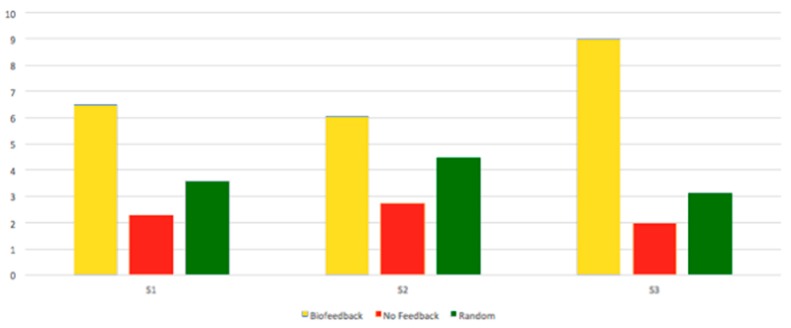
Users’ correct answers for content-based questions after each lecture, Study 2. There is a higher level of correct answers (total number on *Y*-axis) across different sessions for participants who received biofeedback.

Figure 10 shows the users’ test scores for content questions across each session for each type of feedback administered in Study 2. Participants who were provided Biofeedback scored higher (M = 6.865, SD. = 3.06, SE. = 1.02) than participants who were provided random feedback (M = 3.67, SD = 1.92, SE. = 0.641) or no feedback (NF) (M = 2.68, St.d = 0.87, Std.Err. = 0.29). This finding goes in line with Hypothesis 3, whereby haptic feedback improved learning performance of users (retention and comprehension of presented concepts).

### 6.2. Informal Interviews with Users from Study 2

We performed interviews with participants as well. The findings were very similar to ones from Study 1; in particular, on the effectiveness of the feedback, Participants 9 and 11 (biofeedback group) both reported that the “*necklace did help in focusing on the lecture.*”.

The mere presence of the device was also mentioned as possibly helping with engagement (pointed out by two users). Participants had an opportunity to observe the effect from the system over longer period of time than their peers from Study 1. In particular, Participant 8 (biofeedback group) reported: “*It was interesting to see the behavior of the necklace. So last time, when I was attending the lecture two, in the questionnaire there was a question if you feel tired, and I felt that I was not tired, and the necklace only vibrated a couple of times. But then, yesterday evening, I was studying for my finals, and I went to bed very late and today I was not in the right state of the mind, and it* [scarf] *hit me 6 or 7 times. It really got me, it was really accurate.*”.

Several participants reported not to perceive an EEG band as a socially user-friendly wearable device; for example, Participants 2, 3, and 4 (random feedback group) reported that the “*EEG band was not usual to wear and* [they were] *not sure if* [they] *would use it in social environment.*” Participant 5 (no feedback group) pointed out that “*I would wear the system in social environment if everyone else would wear it as well.*”.

We further discuss these comments for both studies as well as their implications for future form factors of the system in the next section.

### 6.3. Conclusions for Study 2

Although the number of participants was not large, if we were to draw some conclusions, our first and third hypotheses—whereby that haptic feedback will increase EEG-based engagement score and improve learning performance—were not rejected in the experiment. As seen from the results, the EEG-based engagement score of the users who received biofeedback was significantly higher than for those who received either random or no feedback. Our second hypothesis, whereby participants would positively perceive the haptic feedback when evaluating the system, was not rejected either, as we can observe from the subjective questionnaires, informal interviews, and comments from the participants. We further discuss the implications of this study in next section.

## 7. Discussion, Challenges, Future Work, and Limitations

Our first and third hypotheses, whereby haptic feedback in the form of vibrations will increase EEG-based engagement and improve momentary learning performance (see our disclaimer in Section 3.3), were not rejected in our experiments. As seen from the results, the EEG-based engagement score of the users who received biofeedback was significantly higher than those who received either random or no feedback. Our second hypothesis, whereby participants will positively perceive haptic feedback when evaluating the system, was also not rejected in Studies 1 and 2, based on the data collected from the questionnaires, informal interviews, and comments from the participants.

We do not envision AttentivU as a system to be worn 24/7 in order to keep a person continuously attentive or engaged as a continuous high level of engagement can negatively affect learning outcomes [43]. AttentivU might provide an alternative option for students or other users who have problems with staying on task when attention is required, e.g., when attending a lecture. If we are successful with further improvements of the system, AttentivU could become as simple to use as eyeglasses: a solution to improve the mental focus of users when they desire to be more attentive.

We would like to acknowledge several challenges and limitations to be addressed in follow-up projects.

*Challenge 1: Design considerations and form factors.* The EEG headband form factor received mixed reviews from participants in both studies. Although most of them (29/36, Study 1 and 7/12, Study 2) did consider using it on an occasional basis, they mentioned that a slimmer and less heavy headband would be desirable. Although the scarf received positive feedback, we will investigate using other, more compact form factors, like a magnetic clip, so that a person can choose where he/she receives the feedback (e.g., upper part of the body, or not) and whether it is hidden under clothing or not.

Regarding the EEG band, as it received worse feedback from the users, we plan to focus on improving this part of the system as well. We will perform tests with other form factors including around-ear EEG, but we also plan to include other modalities to monitor engagement, for example, eye movements (for example, using electrooculography or EOG). Recently, a paper was presented, where a pair of glasses which combines both EEG and EOG as well as a feedback component within one piece of hardware was developed [44]. Although this new form factor is yet to be tested in learning environments, there is a possibility that combining both EEG and EOG signals to monitor engagement might improve the obtained data and algorithms and help with reducing motion artefacts. Other research prototypes include EEGlass by Vouvopoulos et al. [45] or the EmotiGO glasses research prototype [46], designed for acquisition of a set signals including electrodermal activity, photoplethysmography (PPG), and skin temperature. These signals could be used to infer the emotional states of the user. Other works investigated the possibility of extracting physiological signals like heart rate and breathing rate from Google Glass [47,48,49].

In addition, there are two commercially available pairs of glasses that use either EEG electrodes or EOG electrodes: Smith Lowdown Focus Eyewear Glasses [50] by Muse and JINS MEME glasses [51,52]. Smith Lowdown Focus Eyewear is a pair of EEG glasses with two dry electrodes around the ears on TP9 and TP10 according to the 10–20 EEG positioning system. JINS MEME glasses have EOG sensors on the nose pads. However, both Smith Focus and JINS Meme use a phone to get feedback about person’s state of attention (JINS Meme) or during meditations (Smith glasses). Such feedback limits possible use cases and presents potential privacy issues for users but nevertheless suggests interesting future work directions. Kosmyna et al. [53], for example, used the Smith Focus glasses applied to video learning scenario in a similar manner as in this paper, Study 1.

*Challenge 2: Motion artefacts.* The current prototype, as many others, uses a consumer-based EEG headband. This type of bands is affordable and relatively cheap (between $200 and $500 per unit, which might be useful for multiuser set-up as done in this paper, Study 2). These bands are also simple in set-up and use. Their general usability is described in depth in the literature. The preprocessing and signal processing pipelines were kept as close to the previously published works as possible, to ensure reproducibility of our work. Moreover, the same engagement index and signal processing pipelines were applied in a mobile context in several papers [1,24,35]; thus, our results may remain valid when there is movement. As we performed our experiments in a seated context, big movements were not expected.

*Challenge 3: Set-up.* Though we believe that we did our best to reproduce an online learning condition for our first study and an actual lecture condition for the second one, there are limits to the realism of our experiments, as we wanted to control for several sources of noise, such as extensive movements. For example, we did not let our participants take notes and we also did not allow them to ask questions during the lectures. These constraints are not typical of real-life or online lectures. We plan to test the system in actual classrooms, where current pedagogy is moving away from lecture-style teaching (which is targeted in the current experiments), toward more engaging and interactive learning experiences. We might start with the scenarios where users would be able to take notes and/or talk, before trying to engage in any active movement scenarios.

As future work, for Study 2, an approach from DiLascio et al. [54] could be implemented; the data could be gathered from multiple lectures and teachers to contribute to the generalizability of the study results.

Additionally, with respect to the disclaimer regarding the learning performance in our studies: in this paper we meant “momentary learning performance”, meaning immediate results after the information was presented to a person, in contrast with a definition of the learning performance as measuring “learning gains over time” [42]. In our studies, we did not perform tests again on the same content over a longer period of time, thus all cases of learning performance we mentioned here are referred to as “momentary” ones. It would be interesting to access learning performance over several weeks, months, or even a semester.

Another detail to take into consideration is that users knew they were being monitored, which might have affected their engagement. Seven users reported that they were aware of it and that it made them pay closer attention. However, that is why we included a random and no feedback condition as well.

*Challenge 4: Engagement detection.* Although pilot studies and calibration for each participant prior to each experiment were performed, and the engagement formula used in this work is well documented, additional research is needed and new algorithms could be proposed for detecting engagement level. There is a possibility that by incorporating daily mood and other factors that might have an effect on engagement index in the protocol, its precision would only increase, as it will be more dynamic to capture more facets of engagement and disengagement. A similar approach was also suggested in [1,23]. It would be interesting to see if the choice of different subsets of features could be derived and if it could influence the performance of different classifiers, in line with DiLascio et al. [54]. Additionally, based on the emerging work mentioned in *Challenge 1*, other physiological modalities like EOG could be used to deal with fatigue either offline (research paper using JINS Meme for example, [55]), as well as to measure it in real time combined with EEG by Kosmyna et al., [55].

In future studies, we are also interested in testing other system adaptations and feedback options for closing the loop using AttentivU, for example, by considering an adaptive interface, where the video could be slowed down in the case of low engagement detection or where suggestions for additional information would appear in the end of the video regarding the parts of the video where the user was really engaged with the content of this video. Such adaptations would also go in line with classic adaptive BCI interfaces.

We are also interested in verifying if longer-term use of the system will result in improved engagement level when the system is no longer in use and to what extent, if any, habituation may occur.

## 8. Ethical Considerations

The ultimate goal of this research is to give people a new tool for improving their ability to sustain attention. To date, we have demonstrated that in the short term—immediately after biofeedback was administered—the individual’s engagement is improved. We have yet to evaluate whether this implies that their “reserve of attention” is depleted more quickly, and we also have yet to study what the long-term consequences of using this type of system are: Does longer-term use of the system improve or worsen people’s natural ability to sustain attention? We plan to study these questions in future work. If the system ultimately proves to have the desired outcomes, our hope is that no one will be forced to use this system, whether in work or school settings, but that instead individuals will have the freedom and control to decide when and where they want to use the device. While the prototype discussed in this paper is very visible to others and not very easy to take on or off, since then, we have improved the form-factor by building the same functionality into a set of glasses [44], so that people can use this feedback system privately and discreetly whenever they desire. With respect to privacy, while the system discussed here collects data for analysis and research purposes (all within Institutional Review Board (IRB) regulations), the intended “real-world” version of this system would maximize privacy of the collected data by processing all data locally, rather than sharing or storing them for later review. Lastly, while to date, we have only performed user experiments with neurotypical adults, in the future, we plan to do experiments with minors as well as with populations with ADHD. We plan to fully engage these populations, their families, teachers, and therapists to better understand the sensitive issues involved in the real-world use and consequences of this type of technology.

## 9. Conclusions

Information about a person’s engagement and attention might be a valuable asset in many settings, including work situations, driving, and learning environments. To this end, we propose the first prototype of a device called AttentivU—a wearable system which consists of two main components. Component 1 is represented by an EEG headband used to measure the engagement of a person in real time. Component 2 is a scarf which provides subtle, haptic feedback (vibrations) in real time when a drop in engagement is detected. We tested AttentivU in two separate studies with 48 adults. The participants were engaged in a learning scenario of either watching three video lectures on different subjects or participating in a set of three face-to-face lectures with a professor. There were three feedback conditions administered during both studies: (1) biofeedback, during which the scarf vibrated each time a drop in engagement was detected; (2) random feedback, where the vibrations from the scarf did not correlate or depend on the engagement level detected by the system, and (3) no feedback, when no vibrations were administered. The results show that the biofeedback condition redirected the engagement of the participants to the task at hand and improved their performance on comprehension tests. We anticipate that with further improvements in hardware and signal processing as well as by adequately addressing the ethical questions like data privacy, AttentivU might be relevant and useful for school and college use, workforce use or even in-car driving scenarios as well as other situations where engagement and attention are critical.

## Figures and Tables

**Table 1:** Summary of the statistical tests for Study 2. NF—no feedback.

Random Effect	Variance Explained	Standard Deviation
Slide Number	0	0
Slide Type	0.05	2.19
Vibration Count	0.09	5.36
ANOVA *p* < 0.001		
AIC = 2401.4	BIC = 2724.7	Chi sq. = 248.4
D.f. = 2	Marginal R^2^ = 0.40	Conditional R^2^ = 0.55
Post-hoc (Adjusted Holm), *p* < 0.01 for all		
Pair	Standard Error	z
*Random vs. NF*	1.48	−7.93
*Biofeedback vs. NF*	1.7	14.68
*Random vs. Biofeedback*	2.08	−6.38

## References

[1] Hassib M., Schneegass S., Eiglsperger P., Henze N., Schmidt A., Alt F. (2017). EngageMeter: A system for implicit audience engagement sensing using electroencephalography. Proceedings of the 2017 CHI Conference on Human Factors in Computing Systems (CHI 2017).

[2] Silveira F., Eriksson B., Sheth A., Sheppard A. (2013). Predicting audience responses to movie content from electro-dermal activity signals. Proceedings of the 2013 ACM International Joint Conference on Pervasive and Ubiquitous Computing (UbiComp 2013).

[3] Vi C.T., Takashima K., Yokoyama H., Liu G., Itoh Y., Subramanian S., Kitamura Y. (2013). D-FLIP: Dynamic and Flexible interactive PhotoShow. International Conference on Advances in Computer Entertainment.

[4] Hutt S., Mills C., Bosch N., Krasich K., Brockmole J., D’Mello S. (2017). Out of the Fr-Eye-ing Pan: Towards gaze-based models of attention during learning with technology in the classroom. Proceedings of the 25th Conference on User Modeling, Adaptation and Personalization (UMAP 2017).

[5] Sinatra G.M., Heddy B.C., Lombardi D. (2015). The challenges of defining and measuring student engagement in science. Educ. Psychol..

[6] Abdelrahman Y., Hassib M., Marquez M.G., Funk M., Schmidt A. (2015). Implicit engagement detection for interactive museums using brain-computer interfaces. Proceedings of the 17th International Conference on Human-Computer Interaction with Mobile Devices and Services Adjunct (MobileHCI 2015).

[7] Andujar M., Gilbert J.E. (2013). Let’s learn: Enhancing user’s engagement levels through passive brain-computer interfaces. CHI ’13 Extended Abstracts on Human Factors in Computing Systems (CHI EA 2013).

[8] Oken B.S., Salinsky M.C., Elsas S.M. (2006). Vigilance, alertness, or sustained attention: Physiological basis and measurement. Clin. Neurophysiol..

[9] Kamzanova A.T., Matthews G., Kustubayeva A.M., Jakupov S.M. (2011). EEG Indices to Time-On-Task Effects and to a Workload Manipulation (Cueing). World Acad. Sci. Eng. Technol..

[10] Reinerman L.E., Matthews G., Warm J.S., Langheim L.K., Parsons K., Proctor C.A., Siraj T., Tripp L.D., Stutz R.M. (2006). Cerebral blood flow velocity and task engagement as predictors of vigilance performance. Proc. Hum. Factors Ergon. Soc..

[11] Freeman F.G., Mikulka P.J., Prinzel L.J., Scerbo M.W. (1999). Evaluation of an adaptive automation system using three EEG indices with a visual tracking task. Biol. Psychol..

[12] Fredricks J.A., Blumenfeld P.C., Paris A.H. (2004). School engagement: Potential of the concept, state of the evidence. Rev. Educ. Res..

[13] Reeve J.M., Tseng C.-M. (2011). Agency as a fourth aspect of students’ engagement during learning activities. Contemp. Educ. Psychol..

[14] Shernoff D.J., Csikszentmihalyi M., Schneider B., Shernoff E.S. (2014). Student engagement in high school classrooms from the perspective of flow theory. Applications of Flow in Human Development and Education.

[15] Azevedo R. (2015). Defining and measuring engagement and learning in science: Conceptual, theoretical, methodological, and analytical issues. Educ. Psychol..

[16] Raca M., Kidzinski Ł., Dillenbourg P. Translating head motion into attention-towards processing of student’s body language. Proceedings of the 8th International Conference on Educational Data Mining: International Educational Data Mining Society.

[17] Raca M., Dillenbourg P., Suthers D., Verbert K., Duval E., Ochoa X. (2013). System for assessing classroom attention. Proceedings of the Third International Conference on Learning Analytics and Knowledge (LAK 2013).

[18] Byrne E.A., Parasuraman R. (1996). Psychophysiology and adaptive automation. Biol. Psychol..

[19] Boucsein W., Haarmann A., Schaefer F. (2007). Combining skin conductance and heart rate variability for adaptive automation during simulated IFR flight. Engineering Psychology and Cognitive Ergonomics.

[20] Yuksel B.F., Oleson K.B., Harrison L., Peck E.M., Afergan D., Chang R., Jacob R.J.K. (2016). Learn Piano with BACh: An adaptive learning interface that adjusts task difficulty based on brain state. Proceedings of the 2016 CHI Conference on Human Factors in Computing Systems (CHI 2016).

[21] Berka C., Levendowski D.J., Lumicao M.N., Yau A., Davis G., Zivkovic V.T., Olmstead R.E., Tremoulet P.D., Craven P.L. (2007). EEG correlates of task engagement and mental workload in vigilance, learning, and memory tasks. Aviat. Space Environ. Med..

[22] Frey J., Grabli M., Slyper R., Cauchard J.R. (2018). Breeze: Sharing Biofeedback through Wearable Technologies. Proceedings of the 2018 CHI Conference on Human Factors in Computing Systems (CHI 2018).

[23] Hassib M., Khamis M., Friedl S., Schneegass S., Alt F. (2017). Brainatwork: Logging cognitive engagement and tasks in the workplace using electroencephalography. Proceedings of the 16th International Conference on Mobile and Ubiquitous Multimedia (MUM 2017).

[24] Szafir D., Mutlu B. (2012). Pay attention: Designing adaptive agents that monitor and improve user engagement. Proceedings of the SIGCHI Conference on Human Factors in Computing Systems (CHI 2012).

[25] Huang J., Yu C., Wang Y., Zhao Y., Liu S., Mo C., Liu J., Zhang L., Shi Y. (2014). FOCUS: Enhancing children’s engagement in reading by using contextual BCI training sessions. Proceedings of the SIGCHI Conference on Human Factors in Computing Systems (CHI 2014).

[26] Marchesi M., Riccò B. (2013). BRAVO: A brain virtual operator for education exploiting brain-computer interfaces. Proceedings of the CHI 2013 Extended Abstracts on Human Factors in Computing Systems (CHI EA 2013).

[27] Qian X., Loo B.R.Y., Castellanos F.X., Liu S., Koh H.L., Poh X.W.W., Krishnan R., Fung D., Chee M.W.L., Guan C. (2018). Brain-computer-interface-based intervention re-normalizes brain functional network topology in children with attention deficit/hyperactivity disorder. Transl. Psychiatry.

[28] Sclater N., Peasgood A., Mullan J. Learning Analytics in Higher Education: A Review of UK and International Practice. https://www.jisc.ac.uk/sites/default/files/learning-analytics-in-he-v2_0.pdf.

[29] Re:Vibe. https://revibetech.com.

[30] Mindset. https://www.thinkmindset.com/science/.

[31] BrainCo. https://www.brainco.tech.

[32] Gwin J.T., Gramann K., Makeig S., Ferris D.P. (2010). Removal of movement artifact from high-density EEG recorded during walking and running. J. Neurophysiol..

[33] Zeagler C. (2017). Where to wear it: Functional, technical, and social considerations in on-body location for wearable technology 20 years of designing for wearability. Proceedings of the 2017 ACM International Symposium on Wearable Computers (ISWC 2017).

[34] Karuei I., MacLean K.E., Foley-Fisher Z., MacKenzie R., Koch S., El-Zohairy M. (2011). Detecting vibrations across the body in mobile contexts. Proceedings of the SIGCHI Conference on Human Factors in Computing Systems (CHI 2011).

[35] Pope A.T., Bogart E.H., Bartolome D.S. (1995). Biocybernetic system evaluates indices of operator engagement in automated task. Biol. Psychol..

[36] Molteni E., Bianchi A.M., Butti M., Reni G., Zucca C. Analysis of the dynamical behaviour of the EEG rhythms during a test of sustained attention. Proceedings of the 2007 29th Annual International Conference of the IEEE Engineering in Medicine and Biology Society.

[37] Fairclough S.H., Moores L.J., Ewing K.C., Roberts J. Measuring task engagement as an input to physiological computing. Proceedings of the 2009 3rd International Conference on Affective Computing and Intelligent Interaction and Workshops.

[38] Wilson K., Korn J.H. (2007). Attention during lectures: Beyond ten minutes. Teach. Psychol..

[39] Vi C.T., Alexander J., Irani P., Babaee B., Subramanian S. (2014). Quantifying EEG Measured Task Engagement for Use in Gaming Applications.

[40] Hincks S.W., Bratt S., Poudel S., Phoha V., Jacob R.J.K., Dennett D.C., Hirshfield L.M. (2017). Entropic brain-computer interfaces using fNIRS & EEG to measure attentional states in a Bayesian framework. PhyCS.

[41] Blasco-Arcas L., Buil I., Hernández-Ortega B., Sese F.J. (2013). Using clickers in class. The role of interactivity, active collaborative learning and engagement in learning performance. Comput. Educ..

[42] Yan S., Ding G.Y., Li H., Sun N., Wu Y., Guan Z., Zhang L., Huang T. (2016). Enhancing audience engagement in performing arts through an adaptive virtual environment with a brain-computer interface. Proceedings of the 21st International Conference on Intelligent User Interfaces (IUI 2016).

[43] Kosmyna N., Morris C., Sarawgi U., Nguyen T., Maes P. AttentivU: A wearable pair of EEG and EOG glasses for real-time physiological processing. Proceedings of the 16th IEEE International Conference on Wearable and Implantable Body Sensor Networks (BSN 2019).

[44] Vourvopoulos A., Niforatos E., Giannakos M. (2019). EEGlass: An EEG-eyeware prototype for ubiquitous brain-computer interaction. Proceedings of the 2019 ACM International Joint Conference on Pervasive and Ubiquitous Computing and Proceedings of the 2019 ACM International Symposium on Wearable Computers.

[45] Saadatzi M.N., Tafazzoli F., Welch K.C., Graham J.H. EmotiGO: Bluetooth-enabled eyewear for unobtrusive physiology-based emotion recognition. Proceedings of the IEEE International Conference on Automation Science and Engineering (CASE).

[46] Dementyev A., Holz C. (2017). DualBlink: A wearable device to continuously detect, track, and actuate blinking for alleviating dry eyes and computer vision syndrome. Proc. ACM Interact. Mob. Wearable Ubiquitous Technol..

[47] Hernandez J., Li Y., Rehg J.M., Picard R.W. (2015). Cardiac and Respiratory Parameter Estimation Using Head-mounted Motion-sensitive Sensors. EAI Endorsed Trans. Pervasive Health Technol..

[48] Hernandez J., Picard R.W. (2014). SenseGlass: Using google glass to sense daily emotions. Proceedings of the Adjunct Publication of the 27th Annual ACM Symposium on User Interface Software and Technology (UIST 2014 Adjunct).

[49] Smith LowdownFocus Glasses. https://www.smithoptics.com/us/lowdownfocus.

[50] Uema Y., Inoue K. (2017). JINS MEME algorithm for estimation and tracking of concentration of users. Proceedings of the 2017 ACM International Joint Conference on Pervasive and Ubiquitous Computing and Proceedings of the 2017 ACM International Symposium on Wearable Computers.

[51] JINS MEME Glasses. https://jins-meme.com/en/researchers/.

[52] Kosmyna N., Sarawgi U., Maes P. (2018). AttentivU: Evaluating the feasibility of biofeedback glasses to monitor and improve attention. Proceedings of the 2018 ACM International Joint Conference and 2018 International Symposium on Pervasive and Ubiquitous Computing and Wearable Computers (UbiComp 2018).

[53] Di Lascio E., Gashi S., Santini S. (2018). Unobtrusive assessment of students’ emotional engagement during lectures using electrodermal activity sensors. Proc. ACM Interact. Mob. Wearable Ubiquitous Technol..

[54] Tag B., Vargo A.W., Gupta A., Chernyshov G., Kunze K., Dingler T. (2019). Continuous alertness assessments: Using EOG glasses to unobtrusively monitor fatigue levels In-The-Wild. Proceedings of the 2019 CHI Conference on Human Factors in Computing Systems (CHI 2019).

[55] Kosmyna N., Morris C., Nguyen T., Zepf S., Hernandez J., Maes P. (2019). AttentivU: Designing EEG and EOG compatible glasses for physiological sensing and feedback in the car. Proceedings of the 11th International Conference on Automotive User Interfaces and Interactive Vehicular Applications (AutomotiveUI 2019).

